# Acetaminophen-Induced Rat Hepatotoxicity Based on M1/M2-Macrophage Polarization, in Possible Relation to Damage-Associated Molecular Patterns and Autophagy

**DOI:** 10.3390/ijms21238998

**Published:** 2020-11-26

**Authors:** Yuka Tsuji, Mizuki Kuramochi, Hossain M. Golbar, Takeshi Izawa, Mitsuru Kuwamura, Jyoji Yamate

**Affiliations:** 1 Veterinary Pathology, Graduate School of Life and Environmental Sciences, Osaka Prefecture University, 1-58 Rinku-Ourai-Kita, Izumisano City, Osaka 598-8531, Japan; yuka.tsubasa@gmail.com (Y.T.); kuramochi.mzk@gmail.com (M.K.); golbar@ru.ac.bd (H.M.G.); izawa@vet.osakafu-u.ac.jp (T.I.); kuwamura@vet.osakafu-u.ac.jp (M.K.); 2 Department of Veterinary and Animal Sciences, Faculty of Veterinary and Animal Sciences, University of Rajshahi, Rajshahi-6205, Bangladesh

**Keywords:** DAMP, acetaminophen, APAP, autophagy, rat liver, M1-/M2-macrophage polarization

## Abstract

Overdose of acetaminophen (APAP), an antipyretic drug, is an important cause of liver injury. However, the mechanism in the rat model remains undetermined. We analyzed APAP-induced hepatotoxicity using rats based on M1/M2-macrophage functions in relation to damage-associated molecular patterns (DAMPs) and autophagy. Liver samples from six-week-old rats injected with APAP (1000 mg/kg BW, ip, once) after 15 h fasting were collected at hour 10, and on days 1, 2, 3, and 5. Liver lesions consisting of coagulation necrosis and inflammation were seen in the affected centrilobular area on days 1 and 2, and then, recovered with reparative fibrosis by day 5. Liver exudative enzymes increased transiently on day 1. CD68^+^ M1-macrophages increased significantly on days 1 and 2 with increased mRNAs of M1-related cytokines such as IFN-γ and TNF-α, whereas CD163^+^ M2-macrophages appeared later on days 2 and 3. Macrophages reacting to MHC class II and Iba1 showed M1-type polarization, and CD204^+^ macrophages tended to be polarized toward M2-type. At hour 10, interestingly, HMGB1 (representative DAMPs) and its related signals, TLR-9 and MyD88, as well as LC3B^+^ autophagosomes began to increase. Collectively, the pathogenesis of rat APAP hepatotoxicity, which is the first, detailed report for a rat model, might be influenced by macrophage functions of M1 type for tissue injury/inflammation and M2-type for anti-inflammatory/fibrosis; particularly, M1-type may function in relation to DAMPs and autophagy. Understanding the interplayed mechanisms would provide new insight into hepato-pathogenesis and contribute to the possible development of therapeutic strategies.

## 1. Introduction

The liver can metabolize various kinds of chemicals. Acetaminophen (APAP) is also metabolized in the liver. The chemical is used as safe and effective antipyretics at therapeutic doses; however, its overdose can be a cause of severe liver injury [1,2,3,4,5,6]. In fact, an overdose is the primary cause of acute liver failure worldwide [2]. Normally, APAP is eliminated by glucuronidation and sulfation. When ingested at large amounts, excess APAP undergoes oxidation to form the highly reactive intermediate *N*-acetyl-p-benzoquinone-imine (NAPQI) by cytochrome P450, particularly with CYP2E1 [7]. NAPQI is not harmful if it combines rapidly with glutathione. However, NAPQI causes the depletion of cellular glutathione, and when hepatic glutathione stores are depleted, NAQPI can lead to cell death/injury of hepatocytes [1,2,3,4,5,7]. The initial hepatocytic damage triggers the activation of innate immune cells within the liver, such as Kupffer cells. In addition to Kupffer cells, monocyte-derived macrophages which can infiltrate into the liver from the blood further contribute to the progression of hepatotoxicity [8,9,10]. The roles of such hepatic macrophages in APAP-induced hepatotoxicity remain to be investigated.

Hepatic macrophages have an important role in homeostasis and lesion development [11]. In the liver, the plasticity may result from changes in activation states of Kupffer cells and blood monocyte-derived infiltrating macrophages [12]. Pathologically, macrophages appearing in lesions are classified as M1-type (classically activated macrophages) and M2-type (alternatively activated macrophages); M1-/M2-macrophages are corresponding functionally to Th1 and Th2, respectively [13]. Macrophages can express complicated functions depending on lesion stages. M1-type is induced by IFN-γ and produces pro-inflammatory factors, such as IFN-γ, TNF-α, IL-1β, and IL-6, as well as reactive nitrogen. On the other hand, M2-type is activated mainly by IL-4, and releases anti-inflammatory factors, such as IL-4, IL-10, and TGF-β1; out of them, TGF-β1 is well known to contribute to tissue remodeling and fibrosis. Such macrophage plasticity is called M1-/M2-polarization [10,12,13,14,15,16,17]. 

The pathogenesis of lesions may be determined by M1-/M2-polarization, of which the balance should depend on microenvironments. In thioacetamide (TAA)-induced rat hepatic lesions, CD68 positive M1-macrophages, and CD163 positive M2-macrophages develop almost simultaneously in the injured area [16]. In addition to CD68 and CD163, in this study, we investigated macrophages with other immunophenotypes (for MHC class II, Iba1, and CD204).

Endogenous danger signal molecules that are released by injured or necrotic cells can trigger macrophage functions; these molecules are called damage-associated molecular patterns (DAMPs). DAMPs act as ligands that can activate cell surface patterns recognition receptors such as Toll-like receptors (TLRs), or receptors for advanced glycation end products (RAGE) [18,19,20]. Particularly, TLRs are expressed by immune cells, such as macrophages [19]. Downstream effects of TLR engagement include the production of pro-inflammatory factors and chemokines, as well as MHC expression which may promote an effective adaptive immune response [1,19,21,22]. DAMPs include the non-histone chromatin-binding protein high-mobility group box 1 (HMGB1), heat-shock proteins (HSPs), and S100 protein family [7,23,24,25]. Autophagy is an evolutionarily and strictly regulated lysosomal pathway for intracellular degradation, contributing to cellular homeostasis or lesion development [7,22,26]. Autophagy begins with the sequestration of cytoplasm into double-membrane cytosolic vesicles called autophagosomes which express LC3 (MAPlLC3: microtubule-associated protein light chain 3). DAMPs released from injured hepatocytes may influence autophagy in hepatotoxicity [6,22,27,28]. It is known that rats are not suitable for experimentally-induced hepatotoxicity by APAP; therefore, mice have been widely used [2]. We succeeded in the induction of hepatotoxicity by APAP using rats, which is the first, to our knowledge. To shed some light on the pathogenesis of rat APAP-induced hepatotoxicity, in this study, we investigated possible pathogenesis focusing on M1-/M2-macrophages, of which functions may be related to DAMPs and autophagy.

## 2. Results

### 2.1. Gross and Histopathological Findings, and Serum Biochemistry

Grossly, in the livers of controls and at hour 10, no pathological changes were seen. On days 1 and 2, the livers of some injected rats had extensive discolored areas indicative of necrosis.

**Figure 1:**
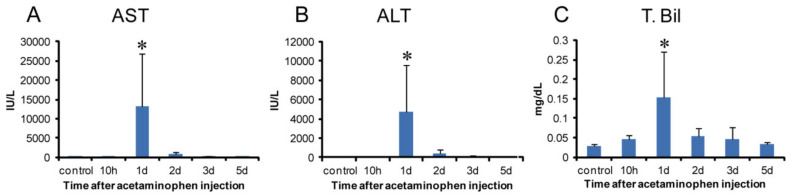
Biochemical analysis in APAP-induced rat hepatotoxicity. Samples were obtained at hour 10, days 1, 2, 3, and 5 after a single injection (1000 mg/kg body weight). The values of aspartate transaminase (AST) (**A**) alanine transaminase (ALT) (**B**) and total bilirubin (T. Bil) (**C**) transiently increased on day 1 with a significant change. Dunnett’s test; *, significantly different from controls at *p* < 0.05. Bar represents mean ± SD.

**Figure 2:**
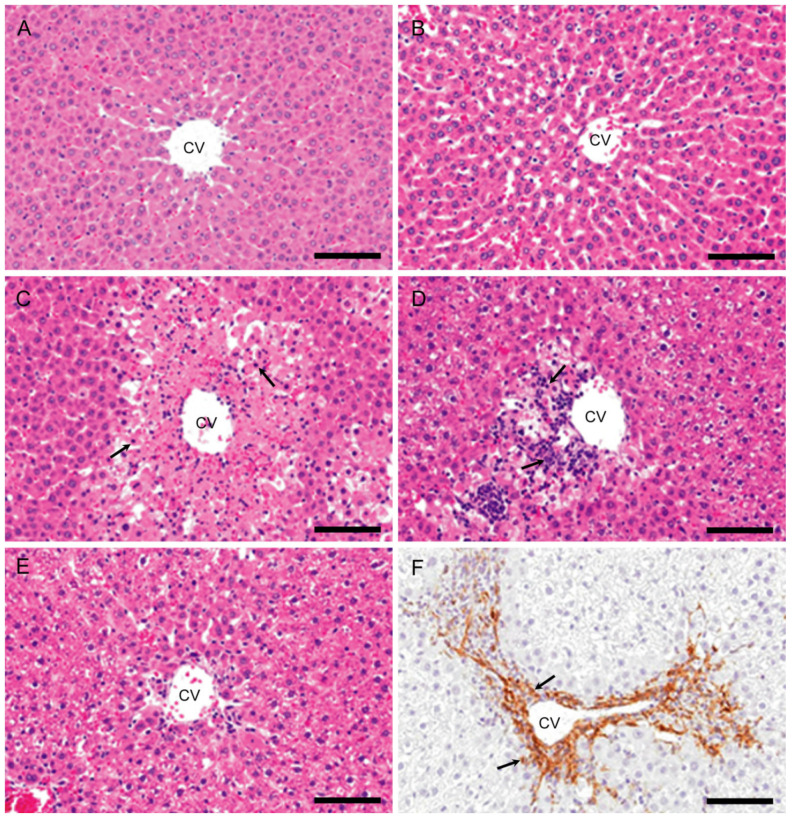
Histopathology of APAP-induced rat hepatotoxicity. In the control (**A**) and at hour 10 (**B**), no significant changes are observed. On day 1, coagulation necrosis of hepatocytes (arrows) accompanied by a small number of inflammatory cells is seen in the affected centrilobular areas (**C**). On day 2, coagulation necrosis with increased infiltration of inflammatory cells (arrows) is seen in the centrilobular areas (**D**). On day 3, the inflammatory cells are almost decreased, and fibrosis develops (**E**). Myofibroblasts immunopositive for α-SMA (arrows) are seen in the fibrotic areas on days 3 (**F**). Hematoxylin and Eosin (**A**–**E**) and immunohistochemical stain, counterstained with hematoxylin (**F**). CV, central vein. Bar = 50 µm.

Biomarker levels for hepatocellular injuries, such as serum AST (Figure 1A), ALT (Figure 1B), and T. Bil (Figure 1C), increased transiently on day 1 with a significant change. In the livers of controls and at hour 10, there were no significant changes in histological architecture in HE-stained sections (Figure 2A,B).

On day 1, coagulation necrosis of hepatocytes accompanied by a small number of inflammatory cells (mainly macrophages and neutrophils) was seen in the affected centrilobular area (Figure 2C), of which lesion development corresponded to the significantly increased serum AST, ALT, and T. Bil. On day 2, coagulation necrosis in the centrilobular area was also seen with more infiltration of inflammatory cells (Figure 2D). On day 3 (Figure 2E), the inflammatory cells were gradually decreased in number, and instead of coagulation necrosis, fibrosis developed; the affected centrilobular areas almost recovered on day 5, indicative of reparative fibrosis. On days 3 and 5, α-SMA positive myofibroblasts, a fibrotic marker [11,29] was seen in the fibrous area with the greatest appearance on day 3 (Figure 2F).

### 2.2. M1-/M2-Macrophages

#### 2.2.1. CD68 Immunostaining for M1-Macrophages

**Figure 3:**
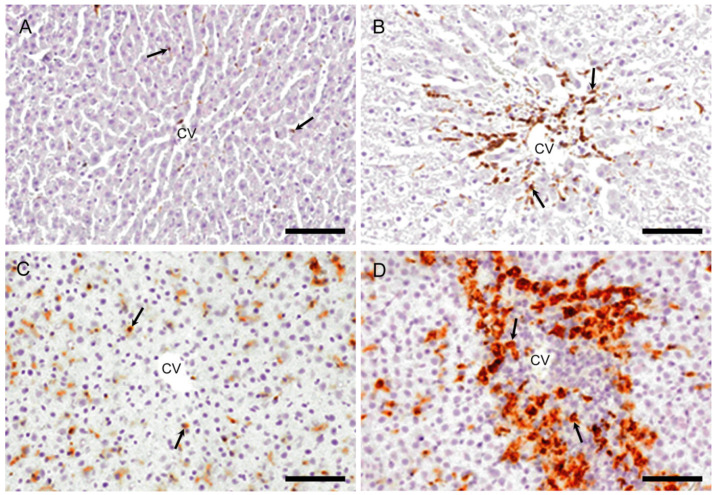
Immunohistochemistry for CD68 (M1-macrophages) and CD163 (M2-macrophages). In controls, very few macrophages positive for CD68 (**A**) are present and cells positive for CD163 (**C**) are seen along the sinusoids (indicative of Kupffer cells). On day 2, the number of macrophages reacting to CD68 (**B**) and CD163 (**D**) is increased, showing different cell shapes to each other. Arrows indicate immunopositive cells. Immunohistochemical staining, counterstained with hematoxylin. CV, central vein. Bar = 50 µm.

**Figure 4:**
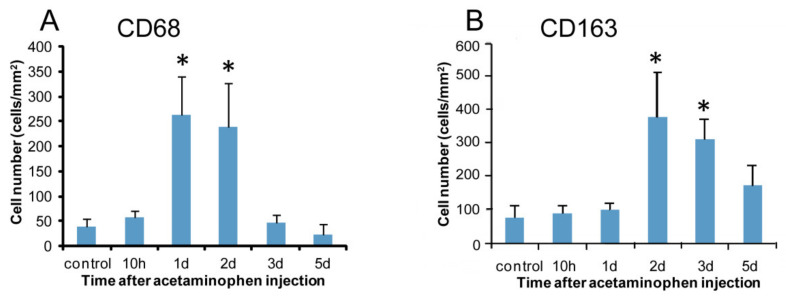
Kinetics of macrophages reacting to CD68 and CD163. Compared to the controls, the numbers of cells expressing CD68 (**A**) on days 1 and 2 and CD163 (**B**) on days 2–3 increase significantly. Dunnett’s test; *, significantly different from controls at *p* < 0.05. Bar represents mean ± SD.

A few CD68 positive macrophages were sporadically seen in the control livers (Figure 3A). At hour 10, the number of CD68 positive cells was similar to the control level. Interestingly, CD68 positive cells were quickly increased significantly on days 1 and 2 (Figure 3B), with a peak on day 1. Thereafter, the CD68 positive cell number on days 3 and 5 was decreased to the control level (Figure 4A). Morphologically, CD68 positive cells were small and round in shape.

#### 2.2.2. CD163 Immunostaining for M2-Macrophages

In the controls, CD163 positive cells were seen along the sinusoid, indicating that they are Kupffer cells (Figure 3C) [30]. At hour 10 and on day 1, the appearance of CD163 positive cells was similar to that of control. CD163 positive cell number showed a significant increase on days 2 (Figure 3D) and 3 with a peak on day 2 (Figure 4B). The number was decreased gradually on day 5; the positive cells were large round or spindle-shaped with enlarged cytoplasm, differing from the shape of CD68 positive cells.

### 2.3. Macrophages with Immunophenotypes for MHC Class II, Iba1 and CD204

#### 2.3.1. MHC Class II Immunostaining

**Figure 5:**
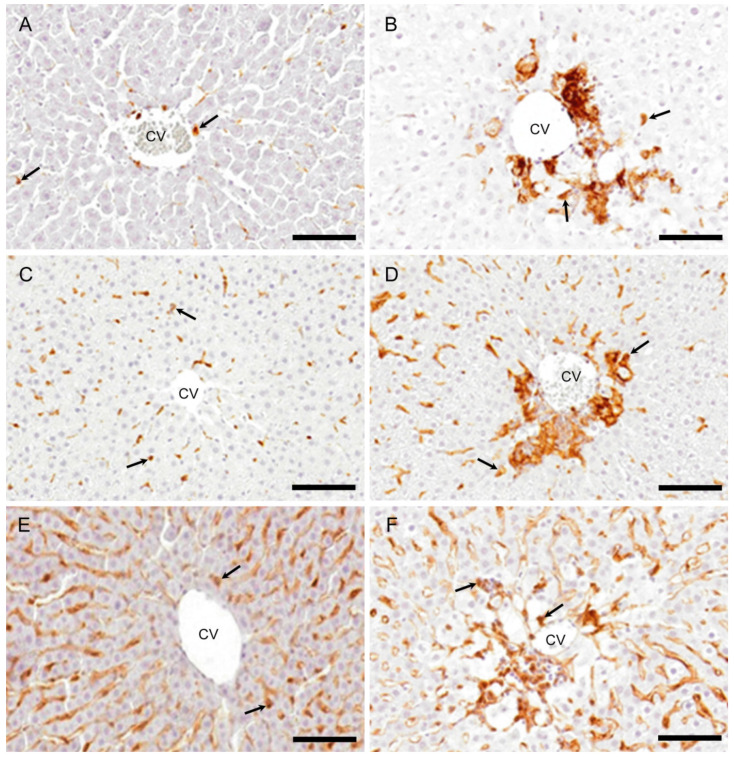
Immunohistochemistry for MHC class II, Iba1, and CD204. Some macrophages are expressing MHC class II (**A**), Iba1 (**C**), and CD204 (**E**) in the centrilobular areas of the control livers. On day 2, the increased numbers of macrophages expressing MHC class II (**B**), Iba1 (**D**), and CD204 (**F**) are seen in the affected centrilobular area. Arrows indicate immunopositive cells. Immunohistochemical staining, counterstained with hematoxylin. CV, central vein. Bar = 50 µm.

**Figure 6:**
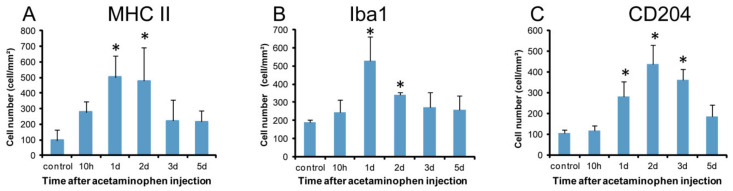
Kinetics of MHC II, Iba1, and CD204 expressing macrophages. Compared to the controls, a significantly increased number of macrophages expressing MHC class II (**A**) on days 1 and 2, Iba1 (**B**) on days 1 and 2, and CD204 (**C**) on days 1–3 are seen. Dunnett’s test; *, significantly different from controls at *p* < 0.05. Bar represents mean ± SD.

In the control livers, MHC class II positive cells were infrequently seen (Figure 5A). There was a tendency to increase in MHC class II positive cell number at hour 10. On days 1 and 2 (Figure 5B), the positive cells were significantly increased, and thereafter, gradually decreased on days 3 and 5 (Figure 6A). Morphologically, the positive cells showed various shapes such as round, large round, ovoid, spindle-shaped, or dendrite.

#### 2.3.2. Iba1 Immunostaining

Iba1 positive cells were sporadically seen along the sinusoids in controls (Figure 5C). At hour 10, the Iba1 positive cell numbers did not show any significant change, but they were significantly increased on days 1 and 2 (Figure 5D), showing a peak on day 1; the appearance was gradually decreased on days 3 and 5 (Figure 6B). Morphologically, the positive cells were similar to MHC class II positive cells, showing various configurations.

#### 2.3.3. CD204 Immunostaining

CD204 positive cells were seen along the sinusoids in the controls (Figure 5E). At hour 10, no significant change was seen in the number. The positive cell number was significantly increased on days 1, 2 (Figure 5F), and 3, showing a peak on day 2, and then, the positive cells were decreased on day 5 (Figure 6C). The cell shapes were similar to those of MHC class II and Iba1.

### 2.4. Double Immunofluorescence for Macrophage Markers

**Figure 7:**
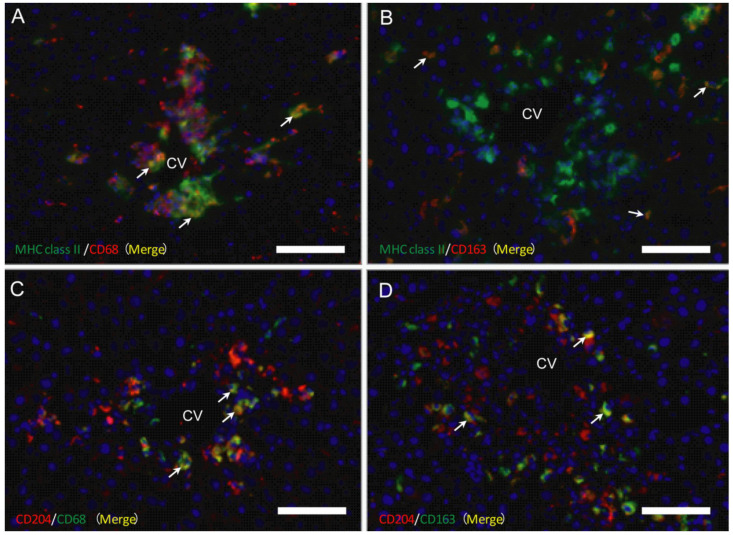
Double immunofluorescence with combinations of MHC class II/CD68 (**A**) and MHC class II/CD163 (**B**) on day 2, and CD204/CD68 (**C**) and CD204/CD163 (**D**) on day 3. Yellow color (arrows) indicates double-positive reactions. Blue is nucleus stained with 4’, 6-diamino-2-phenylindole (DAPI). CV, central vein. Bar = 40 µm.

As mentioned above, CD68 positive cells (for M1-macrophages) appeared in the early injured stages, whereas CD163 positive cells were seen in the later stages than M1-macrophages, indicating the presence of M1-/M2-macrophage polarization. To investigate possible M1-/M2-polarization of macrophages, double immunofluorescence with combinations of MHC class II/CD68 (Figure 7A), MHC class II/CD163 (Figure 7B), CD204/CD68 (Figure 7C), and CD204/CD163 (Figure 7D) was conducted on samples on days 1, 2, and 3.

#### 2.4.1. MHC Class II Positive Macrophages for M1-/M2-Polarization

On days 1, 2, and 3, the percentage of double-positive cells with MHC class II/CD68 (M1) (82%, 79%, and 91%, respectively) to CD68 single-positive cells were more predominant than that of double-positive cells with MHC class II/CD163 (M2) (42%, 53%, and 73%, respectively) to CD163-single positive cells. These findings indicated that MHC class II positive cells showed a tendency toward M1-polarization.

#### 2.4.2. CD204 Positive Macrophages for M1-/M2-Polarization

On days 1, 2, and 3, the percentage of the double-positive cells with CD204/CD68 (M1) (62%, 57%, and 78%, respectively) to CD68 single-positive cells was lower than that of the double-positive cells with CD204/CD163 (M2) (91%, 90%, and 95%, respectively) to CD163-positive cells. These findings indicated the M2-polarization of CD204 positive cells.

### 2.5. CCR2 Positive Macrophages for M1-/M2-Polarization

**Figure 8:**
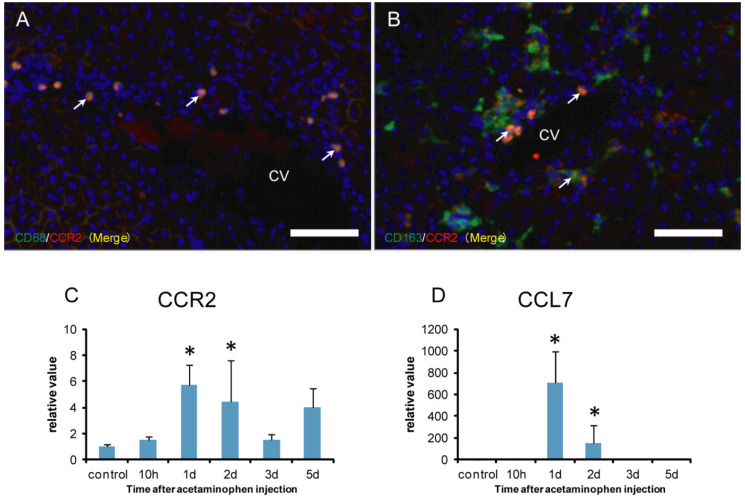
Double immunofluorescence with combinations of CD68/CCR2 (**A**) and CD163/CCR2 (**B**) on day 2, and mRNA expressions for CCR2 (**C**) and CCL7 (**D**) in APAP-induced rat hepatotoxicity. Almost all CD68 positive macrophages label CCR2 (A) (close to 100%), whereas double-positive cells with CCR2/CD163 were very few (**B**) (less than 10%). Yellow color (arrows) indicates double-positive reactions. Blue is nucleus stained with 4’,6-diamino-2-phenylindole (DAPI). mRNA expression levels of CCR2 (**C**) and CCL7 (**D**), normalized by the expression levels of 18s RNA, are significantly increased on days 1 and 2. Dunnett’s test; *, significantly different from controls at *p* < 0.05. Bar represents the mean ± SD. CV, central vein. Bar = 40 µm.

CCR2 is used as a marker of monocyte-derived macrophages [31,32]. To investigate the possible origin of M1-/M2-macrophages, the double immunofluorescence with combinations of CD68/CCR2 (Figure 8A) and CD163/CCR2 (Figure 8B) were conducted. Furthermore, mRNA expressions for CCR2 (Figure 8C) and CCL7 (Figure 8D), a ligand for CCR2, were investigated by the real-time RT-PCR method.

The double immunofluorescence showed that almost all CD68 positive macrophages coexpressed CCR2 (close to 100%) (Figure 8A), whereas approximately 10% of CD163 positive cells reacted to CCR2 (Figure 8B). Additionally, mRNAs of CCR2 (Figure 8C) and CCL7 (Figure 8D) significantly increased on days 1 and 2, corresponding to the increased number of CD68 positive M1 macrophages. Overall, CD68 positive cells might be derived from blood monocytes.

### 2.6. Analysis of M1- and M2-Macrophage-Related Factors

IFN-γ, MCP-1, IL-1β, IL-6, and TNF-α as M1 macrophage-related factors, and IL-4, IL-10, and TGF-β1 as M2 macrophage-related factors were analyzed.

**Figure 9:**
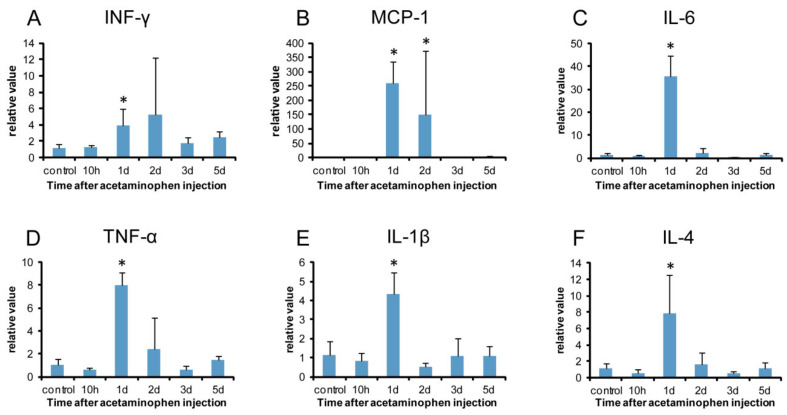
Expressions of mRNAs of M1 macrophage-related (**A-E**) and M2 macrophage-related (**F**) factors. Expression of mRNA levels of IFN-γ (**A**), IL-6 (**C**), TNF-α (**D**), and IL-1β (**E**) show a significant increase on day 1 and that of MCP-1 (**B**) on days 1 and 2. Expression of mRNA level of IL-4 (**F**) significantly increase on day 1. Expression levels were normalized by the expression levels of 18s RNA. Dunnett’s test; *, significantly different from controls at *p* < 0.05. Bar represents the mean ± SD.

mRNAs of IFN-γ (Figure 9A), IL-6 (Figure 9C), TNF-α (Figure 9D), and IL-1β (Figure 9E) showed significantly increased levels on day 1. MCP-1 mRNA significantly increased on days 1 and 2 (Figure 9B). These results indicated that M1 macrophage-related factors increased at the early stages after injection (on day 1), being consistent with the appearance of CD68 positive M1 macrophages with a peak on day 1.

IL-4 mRNA showed a significant increase on day 1 (Figure 9F). Although IL-10 and TGF-β1 mRNAs did not show a significant increase, they tended to increase on days 1 and 2, respectively.

### 2.7. Analysis of DAMPs

#### 2.7.1. HMGB1 Immunostaining

**Figure 10:**
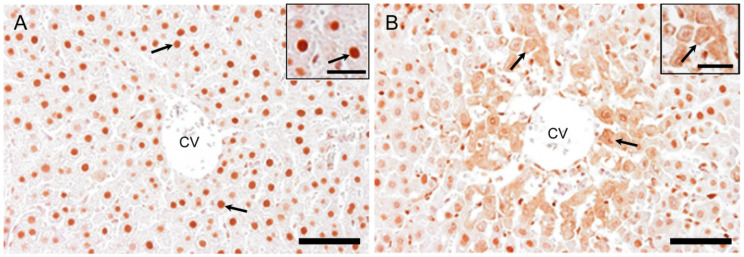
Immunohistochemistry for HMGB1 in APAP-induced rat hepatotoxicity. Nuclei of hepatocytes in the controls are positive for HMGB1 (arrows) at basal level (**A**). Hepatocytes in the affected centrilobular area faintly show cytoplasmic positivity (arrows) with decreased nuclear staining for HMGB1 on day 1 (**B**). Insets are higher magnifications. Immunohistochemical staining. CV, central vein; HMGB1, high mobility group box1. Bar = 50 µm, inset bar = 20 µm.

Nuclei of hepatocytes in the controls were positive for HMGB1 at basal level (Figure 10A). At hour 10, there were some hepatocytes with fine granular reactivity in the cytoplasm in the injured area. On day 1, hepatocytes in the affected centrilobular area showed the greatest cytoplasmic positivity as fine granules and their nuclei looked negative for HMGB1 (Figure 10B). On days 2, 3, and 5, nuclear positivity was the same as that in the controls. These findings indicated that the translocation of nuclear positivity into cytoplasm occurred at hour 10 in some hepatocytes and on day 1 in many injured hepatocytes.

#### 2.7.2. Western Blotting Analysis of HMGB1 and DAMPs Receptors

**Figure 11:**
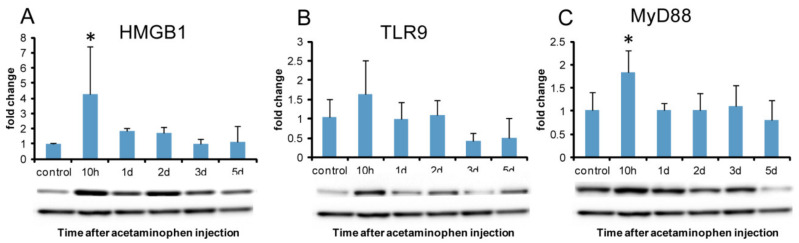
Western blotting for HMGB1, TLR9, and MyD88 in APAP-induced rat hepatotoxicity. The expression level of HMGB1 protein significantly increases at hour 10 (**A**). TLR9 protein expression does not show a significant increase; however, it tends to increase at hour 10 (**B**). MyD88 protein expression significantly increases at hour 10 (**E**). A representative band is shown at each examination point (**A**, **B**, and **C**). GAPDH was used as the loading control (lower panel). Dunnett’s test; *, significantly different from controls at *p* < 0.05. Bar represents mean ± SD. TLR9, toll-like receptor 9; MyD88, Myeloid Differentiation 88.

HMGB1 protein expression showed significantly increased expression at hour 10 when cytoplasmic reactivity began to be seen in hepatocytes (Figure 11A). TLR9 protein expression did not show a significant increase; however, it tended to increase at hour 10 (Figure 11B). TLR2 and TLR4 protein expressions did not show any significant change. Protein expression of MyD88, a central adapter shared between almost all TLRs, significantly increased at hour 10 (Figure 11C), indicating that MyD88-dependent TLR-mediated immune responses were activated at hour 10.

### 2.8. Analysis of Autophagy

To evaluate the autophagy activity, immunohistochemistry and western blotting analyses were performed for LC3B. Western blotting analysis was also performed for RAGE that has been considered to promote autophagy [22].

#### 2.8.1. LC3B Immunostaining for Autophagosome Marker

**Figure 12:**
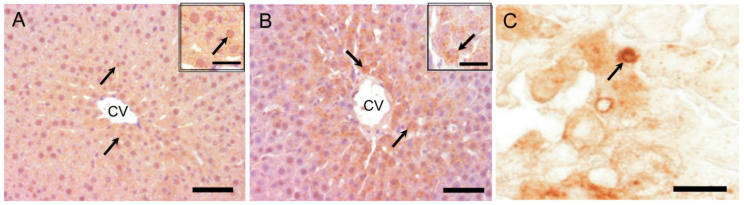
Immunohistochemistry for LC3B in APAP-induced rat hepatotoxicity. LC3B positive cytoplasmic fine granules in hepatocytes (arrows) are sporadically detected at basal level in the control liver (**A**). Compared to the control expression, intracytoplasmic LC3B labeled granules in hepatocytes (arrows) are increased in the centrilobular area with a peak on day 1 (**B**) (Table 1). Abnormally-developed LC3B positive autophagosomes, apparently vacuolated granules, are occasionally seen in some hepatocytes (**C**), suggestive of abnormality of formation (arrow). Insets are higher magnifications. Immunohistochemical staining, counterstained with hematoxylin. CV, central vein; LC3B, microtubule-associated protein light chain 3. Bar = 50 µm (A–B), 15 µm (C), inset bar = 20 µm.

LC3B positive cytoplasmic fine granules in hepatocytes were sporadically detected at basal level in the control livers (Figure 12A). Compared to expression in the controls, cytoplasmic LC3B labeled granules in hepatocytes were more clearly seen in the affected centrilobular area at hour 10 and on day 1 (Figure 12B); particularly, larger cytoplasmic granules reacting to LC3B were occasionally seen on day 1 (Figure 12C), presumably indicating abnormally developed autophagosomes. Furthermore, semi-quantitatively, the degree of LC3B positive cytoplasmic granules in hepatocytes showed a peak on day 1 (Table 1); hepatocytes with cytoplasmic granules were infrequently observed in the centrilobular area on days 2, 3, and 5, returning to the control levels. 

#### 2.8.2. Western Blotting Analysis of Autophagy Marker LC3B and Autophagy-Related Receptor (RAGE)

**Figure 13:**
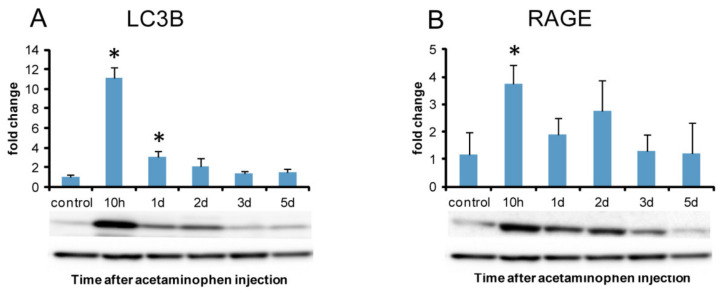
Western blotting analysis of LC3B and RAGE in APAP-induced rat hepatotoxicity. Significantly increased expression of LC3B protein is seen at hour 10 and day 1 (**A**) with a peak on day 10. RAGE protein expression is significantly increased at hour 10 (**B**). A representative band is shown at each examination point (A and B). GAPDH was used as the loading control (lower panel). Dunnett’s test; *, significantly different from controls at *p* < 0.05. Bar represents the mean ± SD. RAGE, receptors for advanced glycation end products.

Consistent with immunohistochemistry for LC3B, the western blotting analysis confirmed the significantly increased expression of LC3B at hour 10 and on day 1, with a peak at hour 10 (Figure 13A). RAGE protein expression significantly increased at hour 10 as the greatest expression of LC3B was seen in immunohistochemistry (Figure 13B).

## 3. Discussion 

APAP-induced hepatotoxicity has been investigated exclusively in mice and some researchers have considered that rats are not suitable for this study [2,33]. It was reported, although overall APAP metabolism was similar in both mice and rats, that mitochondrial protein adducts were lower in rats and that rats also had less oxidative stress [2,33]. It was hypothesized, therefore, that mitochondrial dysfunction is critical for the development of necrosis after APAP treatment [2]. We tried to establish APAP-induced hepatotoxicity in rats. After 15 h of fasting, APAP was injected into F344 male rats at the age of 6 weeks and the obtained centrilobular lesions were almost similar to those in the mice model. In addition to direct hepatic injury by chemicals, generally, macrophages should contribute to the modification of hepatotoxicity [8,9,10]. Since macrophages can show various functions depending on microenvironmental factors, a concept called M1-/M2-macrophage polarization has been proposed in pathological settings [12,34]. Therefore, in this study, we focused on M1-/M2-macrophage functions in relation to DAMPs and autophagy for rat APAP-induced hepatotoxicity. By pathological analyses, it was found that APAP-induced rat liver injury is very complicated.

### 3.1. M1-/M2-Macrophages

M1-macrophages are induced mainly by IFN-γ and produce pro-inflammatory cytokines. Alternatively, M2-macrophages are activated by IL-4 and are associated with the resolution of inflammation, promotion of tissue remodeling, and fibrosis [13]. Immunohistochemically, CD68 and CD163 positive macrophages are regarded as M1- and M2-macrophages, respectively [30]. Additionally, rat macrophages can be also detected with antibodies against MHC class II, Iba1, and CD204 [16,35,36,37].

Macrophages reacting to CD68 (for M1) showed a significant increase on days 1 and 2 following APAP injection; the peak was on day 1 when hepatocyte injury/necrosis was the most prominent, accompanied with a significant increase of AST, ALT, and T. Bil levels. mRNA expressions of IFN-γ, MCP-1, TNF-α, IL-1β, and IL-6 for M1-related factors, which are regarded as pro-inflammatory cytokines [38], also significantly increased on day 1. The appearance pattern of CD68 positive cells corresponded to mRNA expressions of these M1-related factors. These results indicated that M1 macrophages could appear in the early stages, and participate in tissue damage/inflammation via the production of pro-inflammatory factors such as IFN-γ, MCP-1, TNF-α, IL-1β, and IL-6.

Antibody of CD163 labels M2-macrophages [16,39]. In the present study, the number of CD163 positive cells increased on days 2 and 3; the appearance was later than that of CD68 positive M1-macrophages on days 1 and 2. Because M2-macrophages play an important role in reparative fibrosis after tissue injury [38,40,41], fibrotic lesions are seen on days 3 might be related to the appearance of CD163 positive M2-macrophages. Interestingly, the mRNA expression of IL-4 as an M2-macrophage-related factor was transiently increased on day 1. IL-4 is known to induce M2-macrophages [38]. IL-4 might be responsible for further activities of CD163 positive cells because M2-macrophages started to increase from day 2 onwards.

In the present hepatotoxicity, collectively, we demonstrated that M1-/M2-macrophage polarization was clearly present; CD68 positive cells appeared in the early stage as macrophages for tissue damage/inflammation promotion, and thereafter, CD163 positive cells were seen in the late stage in relation to tissue repair/reparative fibrosis. These findings of polarization are different from those seen in TAA-induced rat hepatotoxicity with the simultaneous appearance of M1-/M2-macrophages [16].

In addition to CD68 (for M1) and CD163 (for M2), in the present study, we investigated macrophages with other immunophenotypes (MHC class II, Iba1, and CD204). MHC class II molecules are expressed in antigen-presenting cells such as dendritic cells [21,42]. Iba1 is a cytoplasmic, calcium-binding, inflammation-responsive scaffold protein [36]; it is used as a marker of activated macrophages [36]. CD204 is a class A macrophage scavenger receptor, playing important roles in host defense mechanisms [37]. In the present hepatotoxicity, the number of macrophages reacting to MHC class II and Iba1 showed a significant increase on days 1 and 2 in the centrilobular lesion. The double immunofluorescence revealed that there were greater numbers of CD68 positive cells reacting simultaneously to MHC class II on days 1 to 3, in contrast to the number of MHC class II/CD163 double-positive cells. Therefore, MHC class II positive cells may have a predisposition towards M1-polarization. Double immunofluorescence using Iba1 antibody for M1-/M2-polarization could not be conducted, because Alexa 568-conjugated secondary antibody is not available. However, the expression pattern of Iba1 positive cells was similar to that of CD68 positive M1-macrophages with significantly increased levels on days 1 and 2, suggesting that Iba1 positive macrophages might play a role as M1-macrophages. 

CD204 positive macrophages showed a significant increase on days 1 to 3; on days 2 and 3, the significantly increased number was corresponding to that of CD163 positive M2-macrophages. On day 3, further, although CD68, MHC class II, and Iba1 positive macrophages had already decreased, a significantly increased number of CD204 positive cells were still seen. In the double immunofluorescence, on days 1 to 3, many CD163 positive cells were reacting simultaneously to CD204, being a much greater number than that of CD204/CD68 double-positive cells. Therefore, in the present hepatotoxicity, CD204 positive cells may have a predisposition towards M2-polarization. Presumably, CD204 positive M2-macrophages that appeared on day 1 might be a source of production of M2-related factors, in particular IL-4 that showed a significant increase on day 1 and is considered to be an inducer of M2-macrophages.

M1-polarization of MHC class II positive cells and M2-polarization of CD204 positive cells have been reported in TAA-induced rat hepatic injury [16]. The analysis of M1-/M2-macrophage polarization should be useful to find out the pathogenesis of chemically-induced liver damages.

### 3.2. DAMPs

Damaged/dying cells may release endogenous ligands called DAMPs, and DAMPs can activate pattern recognized receptors, such as TLRs and RAGE [23]. TLRs, widely expressed on leukocytes, regulate innate and adaptive immune responses through the inflammatory cytokines produced by inflammatory cells [19,24,25]. RAGE is the receptor for advanced glycation end-products [18].

HMGB1 is a member of the high mobility group nuclear protein family and well known as DAMPs. HMGB1 protein is a nuclear DNA binding protein [43]. Under normal circumstances, HMGB1 is present in the nuclei, playing important roles in biological processes including transcription and DNA repair [43]. In response to appropriate stimuli, HMGB1 translocates from the nucleus to cytoplasm, thereafter, picked up into secretory lysosomes, and then, is secreted from the cell through exocytosis. HMGB1 released from necrotic/injured cells stimulates monocytes/macrophages through the cell-surface receptors such as TLR2, TLR4, TLR9, and RAGE [44,45,46,47]. It is reported that, in cell signals relating to HMGB1, the translocation of cytoplasmic NF-κB into the nucleus can induce an inflammatory response [44,45,46,47].

In the present hepatotoxicity, immunohistochemically, HMGB1 positivity was seen in the nuclei in the control hepatocytes and began to be seen sporadically as intracytoplasmic fine granules with greater nuclear reactivity in the centrilobular area at hour 10 when a hepatic injury could not be still detected. The peak of cytoplasmic HMGB1 positivity was on day 1 when corresponded to the greatest hepatic injury with a large number of M1-macrophages. HMGB1 protein expression revealed a significant increase at hour 10. Collectively, these findings indicated possible participation of HMGB1 in the development of APAP-induced rat liver lesions to stimulate tissue damage/inflammation which should be due to the participation of M1-macrophages.

Although TLR2/4 are reported as receptors for HMGB1 [44,45,46,47], the protein expression of TLR2 and TLR4 did not show a significant increase in the present study. Instead of these receptors, the protein of TLR9 and RAGE showed a tendency to increase at hour 10, being corresponding to the significantly increased level of HMGB1 protein and MyD88 protein. TLR9 and RAGE might be expressed as DAMPs receptors in the early stage of tissue damage/inflammation in the present hepatotoxicity.

It is interesting to note that MHC class II-expressing cells with the polarization of M1-macrophages appeared exclusively on days 1 and 2. MHC class II molecule is expressed in immune cells such as macrophages through TLRs binding to DAMPs [21]. Because MHC class II-expressing macrophages participate in complicated immune response, it may be interesting to carry out studies on the relation between the immune cells and DAMPs in this hepatotoxicity [48,49,50]. Along with HMGB1, furthermore, S100A4 and HspA1B are involved in DAMPs [49,50]. mRNAs of S100A4 and HspA1B increased on days 1 and 2, and on day 1 onwards, respectively. Various kinds of DAMPs might contribute to the present hepatotoxicity. The interaction of these DAMPs should be investigated in future studies. 

### 3.3. Autophagy

Autophagosome formation is an attempt by cells to limit the spread of subcellular damage by walling off the damaged areas [51]. Autophagy plays an important role in a wide range of diseases [7,26,52]. In drug-induced liver injury, autophagy may contribute not only to homeostasis but also to the suppression of injury [26]. LC3 (microtubule-associated protein 1A/1B light chain 3), the most commonly monitored autophagy marker, has four isoforms; out of them, LC3B is often used for analyses of autophagy function [7]. Immunohistochemically, LC3B-positive fine granules were seen in the cytoplasm of hepatocytes at hour 10, and on day 1, the positivity was the greatest in hepatocytes around the necrotic area; there were some hepatocytes with abnormally-developed granules reacting to LC3B. Furthermore, it was confirmed that LC3B protein expression level was the greatest at hour 10. These findings suggested that the autophagy already occurred functionally at hour 10 before tissue injury on days 1 and 2.

HMGB1 regulates cellular processes such as autophagy. Particularly, HMGB1 is important for oxidative stress-mediated autophagy induction [44]. RAGE, known as a promoter of inflammation via NF-κB, has functions to promote autophagy [22]. Fundamentally, it is considered that autophagy inhibits inflammation via down-regulating caspase 1-dependent inflammasomes which activate IL-1β, showing a critical regulatory function in macrophage polarization that down-regulates inflammation [53,54]; these macrophages may be regarded as M2 type. Based on the information, in the early stages at hour 10 and day 1 in the present hepatotoxicity, the increased autophagy might act as cellular protection to injury, in relation to increased HMGB1 and RAGE. However, HMGB1 released from necrotic/injured cells stimulates monocytes/macrophages through the cell-surface receptors [44,46,47]. The subtle relationship between autophagy and HMGB1 (as DMAPs) may be important in the present hepatotoxicity.

## 4. Materials and Methods

### 4.1. Animals

Five-week-old twenty-seven male F344/DuCrj rats (Charles River Japan, Yokohama, Japan) were used. They were housed in an animal room under controlled temperature (22 ± 3℃) and with a 12 h light-dark cycle. Animals were given a standard commercial diet (DC-8, CLEA Japan Inc., Tokyo, Japan) and tap water *ad libitum*. They were kept for one week to acclimatize to the environment. Twenty-three rats were fasted for 15 h and then injected intraperitoneally once by APAP (Sigma Aldrich Co., Darmstadt, Germany) dissolved in 0.5% metolose (methylcellulose; Shin-Etsu Chemical Co., Ltd, Tokyo, Japan) in distilled water at a dose of 1000 mg/kg body weight to induce liver injury. Rats were fed one day later and were sacrificed under deep isoflurane anesthesia at 10 h, and on 1, 2, 3, and 5 days (n = 4 or 5 at each point) after APAP injection. The remaining four rats served as controls were injected 0.5% metolose in distilled water in the same way, and sacrificed on day 5. The dose was determined based on data in our preliminary experiments with different doses, along with information in a previous article [2] 

The experimental protocols and animal housing conformed to the institutional guidelines of Osaka Prefecture University for the Care and Use of Experimental animals, were approved by the ethical committee of the University and were registered on Osaka Prefecture University Care and Use of Experimental animals register (28-12, 1 April 2016; 29-5, April 1 2017; 30-2, April 1 2018 and 19-51, April 1 2019).

### 4.2. Serum Biochemistry

Blood samples were collected from the abdominal aorta and separated sera were subjected to biochemical analyses of aspartate transaminase (AST), alanine transaminase (ALT), and total bilirubin (T. Bil) (SRS Inc., Tokyo, Japan).

### 4.3. Histopathology and Immunohistochemistry

Tissues from the left lateral lobe of the livers were fixed in 10% neutral buffered formalin, or periodate-lysine-paraformaldehyde (PLP) solution. Formalin solution-fixed specimens were processed routinely and embedded in paraffin wax. PLP solution-fixed specimens were embedded in paraffin with the AMeX method [16]. The fresh liver tissues were also embedded in Tissu Mount^®^ (Chiba Medical, Saitama, Japan) and frozen immediately at –80 °C until use.

#### 4.3.1. Histopathological Examination

Sections were cut at 3 μm in thickness and were stained with hematoxylin and eosin (HE) for morphological observations. 

#### 4.3.2. Immunohistochemistry

Sections cut at 3 μm were subjected to immunohistochemical staining with primary antibodies specific for rat macrophages such as CD68 (M1-macrophages), CD163 (M2- macrophages), MHC class II, Iba1 and CD204: for myofibroblasts (α-SMA): for autophagosomes (LC3B): for HMGB1 (a representative DAMPs). Detailed information on these antibodies is summarized in Table 2. PLP- and formalin-fixed, dewaxed sections were pre-treated with 5% skimmed milk in phosphate-buffered saline (PBS) for 10 min and allowed to react with primary antibodies for 1 h at room temperature. After incubation in 3% H_2_O_2_ for 15 min, they have incubated with horseradish peroxidase-conjugated secondary antibody (Histofine Simple Stain MAX PO^®^; Nichirei Biosciences, Tokyo, Japan) for 1 h. Positive reactions were visualized with 3, 3′-diaminobenzidine (DAB Substrate Kit; Nichirei Biosciences, Tokyo, Japan). Sections were lightly counterstained with hematoxylin.

#### 4.3.3. Double Immunofluorescence

Fresh frozen sections (10 µm in thickness) were used for double immunofluorescence staining. Sections were fixed in acetone:methanol mixed solution (1:1) at 4 °C for 15 min except for double immunofluorescence with CD204/CD68 or CD204/CD163. For the CD204/CD68 or CD204/CD163 staining, sections were fixed in PLP solution at 4 °C for 15 min. All sections for double immunofluorescence were dried for 30 min at room temperature, followed by blocking with 10% normal goat serum for 30 min at room temperature. Thereafter, they were incubated with the primary antibodies at 4 °C overnight; after washing in PBS, they were incubated with the secondary antibody for 45 min at room temperature: Alexa 568/anti-mouse IgG1 (Invitrogen Co., CA, USA; ×500). For double immunofluorescence with CD204/CD68 or CCR2/CD68, sections were incubated with the labeled-CD68 antibody (Mouse anti-rat CD68: Alexa Fluor^®^ 488, AbD Serotec, Oxford, UK). The labeled-MHC class II antibody (Mouse anti-rat MHC Class II H-2I-Ak/s: Alexa Fluor^®^ 488, AbD Serotec, Oxford, UK) was used for the staining with MHC class II/CD68 or MHC class II/CD163. Sections stained with CD204/CD163 or CCR2/CD163 were incubated with the labeled-CD163 antibody (mouse anti-rat CD163: FITC, AbD Serotec, Oxford, UK). All labeled antibodies were reacted for 45 min at room temperature. After washing in PBS, sections were mounted with SlowFade^®^ Gold antifade reagent with 4’, 6-diamino-2-phenylindole (DAPI; Vectashield^®^; Vector Laboratories, CA, USA) for nuclear stain. Signals were detected by VS120 Virtual Slide System (Olympus, Tokyo, Japan).

### 4.4. Real-Time Reverse Transcriptase-Polymerase Chain Reaction (RT-PCR)

To evaluate the expression of M1- or M2-macrophage-related factors, and chemokines such as CCR2 and CCL7, real-time RT-PCR was performed. Liver tissues from the left intermediate lobe were immediately soaked in RNA later^®^ (Qiagen GmbH, Hilden, Germany) and stored at –80 °C. Total RNA was extracted by using an SV Total RNA Isolation System (Promega, WI, USA). Total RNA was reverse-transcribed to cDNA using Super Script^®^ VILO ^TM^ cDNA Synthesis Kit (Invitrogen Co., CA, USA).

For quantification of IL-6, IFN-γ, MCP-1, TNF-α, IL-1β, IL-4, IL-10, TGF-β1, CCR2, and CCL7, Thunderbird^®^Probe qPCR Mix (Toyobo, Co., Ltd., Osaka, Japan) were used with TaqMan Gene Expression Assays (Life Technologies, Massachusetts, MA, USA) (Table 3). The amplification program consisted of 1 cycle at 95 °C with a 1 min hold followed by 40 cycles at 95 °C of denaturing temperature with a 15 s hold, 60 °C of extension temperature with a 30 s hold, and then 20 °C of cooling with a 10 s hold. The expression values of target genes were normalized by the expression values of 18s rRNA.

### 4.5. Cell Counts

The number of CD68, CD163, Iba1, MHC class II, and CD204 positive cells in centrilobular areas of the hepatic lobule was counted using WinROOF software (Mitani Corp., Fukui, Japan) and expressed as the number of positive cells per unit area (cells/mm^2^) [16].

For double-labeled macrophages for MHC class II^+^/CD68^+^, MHC class II^+^/CD163^+^, CD204^+^/CD68^+^, and CD204^+^/CD163^+^, as well as CD68^+^/CCR2^+^ and CD163^+^/CCR2^+^, the number of single- and double-positive cells were counted with VS120 Virtual Slide Microscope System (Olympus, Tokyo, Japan) [16].

### 4.6. Western Blot

Liver samples from the left intermediate lobes were homogenized in RIPA buffer (20 mM Tris-HCl pH 7.5, 150 mM NaCl, 1 mM EDTA, 1 mM EGTA, 1% NP-40, 0.1% deoxycholate, 0.1% SDS, 1 mM NaF, 0.1 mM Na_3_VO_4_, 1 mM PMSF and proteinase inhibitor cocktail; Nacalai Tesque, Kyoto, Japan). After centrifugation at 13,000 × g for 10 min, the supernatant was mixed with an equal volume of 2×SDS sample buffer (125 mM Tris-HCl pH6.8, 4% SDS, 30% glycerol, and 10% 2-mercaptoethanol) and then boiled at 95 °C for 5 min. Samples were separated on 5–20% polyacrylamide gels and transferred to polyvinylidene difluoride (PVDF) membranes (BioRad, CA, USA). Membranes were treated with 5% skimmed milk in phosphate-buffered saline with tween 20 (PBST) for 1 h and then incubated overnight at 4 °C with rabbit anti-HMGB1 (Gene Tex, TX, USA), rabbit anti-TLR2 (BioVision, CA, USA), mouse anti-TLR4 (Novus, Littleton, CO, USA), mouse anti-TLR9 (Novus, Littleton, CO, USA), rabbit anti-MyD88 (Abcam, Cambridge, UK), rabbit anti-LC3B (Sigma-Aldrich Co., St. Louis, MO, USA), rabbit anti-RAGE (Novus, Littleton, CO, USA) and rabbit anti-GAPDH (loading control) antibodies (Sigma-Aldrich Co., St. Louis, MO, USA), followed by incubation with peroxide-conjugated secondary antibody (Histofine Simple Stain, MAX-PO; Nichirei, Tokyo, Japan) for 30 min. Signals were visualized with ECL prime (GE Healthcare, Little Chalfont, UK), and quantified with a luminescent image analyzer (LAS-4000; GE Healthcare, Little Chalfont, UK).

### 4.7. Statistical Analysis

Data are represented as mean ± standard deviation (SD). Statistical analyses were performed using Dunnett’s test. Significance was accepted at *p* < 0.05.

## 5. Conclusions

To shed some light on the pathogenesis of APAP-induced rat liver injury, we focused on the M1-/M2-macrophage polarization concept in relation to the effects of DAMPs and autophagy. M1-macrophages and its related factors increased in the early stages, whereas M2-macrophages subsequently appeared in the late stage. Particularly, HMGB1 worked as one of the DAMPs before tissue injury. HMGB1 binding to TLR9 might initiate inflammation, which should be closely related to the polarization of M1-macrophages. Autophagosomes, demonstrable with LC3B immunohistochemistry and western blot analysis, might take part in the early events via RAGE expression, of which functions might have cytoprotection to injury. Collectively, it was found that the association of M1-/M2-macrophage polarization with DAMPs and autophagy might have been responsible for the pathogenesis of APAP-induced rat liver injury. Understanding the mechanisms of DAMP, autophagy, and M1-/M2-macrophages at molecular levels would provide deeper insight into the hepato-pathogenesis. In addition, to investigate the detailed mechanisms of APAP-induced rat hepatotoxicity at the molecular level, this rat model would be useful for the development of a protective compound for hepatotoxicity in therapeutic strategies. This study is the first report of APAP-induced rat hepatotoxicity with detailed analyses focusing on M1-/M2-macrophage polarization. 

## Figures and Tables

**Table 1:** Semi-quantitative analysis for LC3B immunoexpression in the affected centrilobular area.

control	10 h	1 d	2 d	3 d	5 d
±	＋	＋＋	±	±	±

h, hour; d, day; MAPl-LC3B, microtubule-associated protein light chain 3B. ±, very faintly; ＋, moderately; ＋＋, more greatly.

**Table 2:** Primary antibodies used in immunohistochemistry.

Antibody (Clone)	Host	Fixative	Dilution	Pretreatment	Source
CD68 (ED-1)	Mouse Monoclonal	PLP solution	1/500	10 µg/mL proteinase K, RT, 10 min	Millipore, Massachusetts, USA
CD163 (ED-2)	Mouse Monoclonal	Acetone/methanol (1:1)	1/300	―	AbD Serotec, Oxford, UK
MHC class II (OX-6)	Mouse Monoclonal	PLP solution	1/300	Microwave in Citrate buffer, 20 min	AbD Serotec, Oxford, UK
CD204 (SRA-E5)	Mouse Monoclonal	PLP solution	1/500	Microwave in Citrate buffer, 20 min	Transgenic Inc, Kumamoto, Japan
Iba1	Rabbit Polyclonal	Formalin	1/500	Microwave in Citrate buffer, 20 min	Wako, Osaka, Japan
α-SMA (1A4)	Mouse Monoclonal	Formalin	1/1,000	―	Dako, Glostrup, Denmark
HMGB1	Rabbit Polyclonal	PLP solution	1/200	Microwave in Citrate buffer, 20 min	Abcam, Cambridge, UK
LC3B	Rabbit Polyclonal	PLP solution	1/1,000	Microwave in Citrate buffer, 20 min	SIGMA, St. Louis, USA

MHC class II, major histocompatibility complex II; α-SMA, alpha-smooth muscle actin; HMGB1, high mobility group box 1; PLP, periodate-lysine-paraformaldehyde.

**Table 3:** Real-time RT-PCR probes.

Probe	Assay ID	Probe	Assay ID
IL-6	Rn01410330_m1	IL-10	Rn00563409_m1
IFN-γ	Rn00594078_m1	TGF-β1	Rn00572010_m1
MCP-1	Rn00580555_m1	CCR2	Rn01637698_s1
TNF-α	Rn01525859_g1	CCL7	Rn01467286_m1
IL-1β	Rn00580432_ml	Ribosomal 18s	Hs99999901_s1
IL-4	Rn01456866_m1	-	-

IL, Interleukin; IFN, Interferon; MCP, Monocyte chemoattractant protein; TNF, Tumor necrosis factor; TGF, Transforming growth factor; CCR, Chemokine receptor; CCL, Chemokine ligand.

## Abbreviations

T. Bil	Total bilirubin
ALT	Alanine transaminase
APAP	Acetaminophen
AST	Aspartate transaminase
CCL	Chemokine ligand
CCR	Chemokine receptor
CD	Cluster of differentiation
CV	Central vein
DAMP	Damage associated molecular pattern
ECMs	Extracellular matrices
HMGB	High mobility group box
Iba	Ionized calcium binding adaptor
IFN	Interferon
IL	Interleukin
MCP	Monocyte chemoattractant protein
MHC	Major histocompatibility complex
MyD	Myeloid Differentiation
PLP	Periodate-lysine-paraformaldehyde
RAGE	Receptor for Advanced Glycation End-products
RT-PCR	Reverse transcriptase polymerase chain reaction
SMA	Smooth muscle actin
TAA	Thioacetamide
TGF	Transforming growth factor
TLR	Toll-like receptor
TNF	Tumor necrosis factor
